# Light quality affects the proliferation of in vitro cultured plantlets of *Camellia oleifera* Huajin

**DOI:** 10.7717/peerj.10016

**Published:** 2020-10-06

**Authors:** Chaoyin He, Yanling Zeng, Yuzhong Fu, Jiahao Wu, Qin Liang

**Affiliations:** 1 College of Forestry, Central South University of Forestry and Technology, Changsha, Hunan, China; 2 Key Laboratory of Cultivation and Protection for Non-Wood Forest Trees, Ministry of Education, Central South University of Forestry and Technology, Changsha, Hunan, China; 3 Key Lab of Non-wood Forest Products of State Forestry Administration, Central South University of Forestry and Technology, Changsha, Hunan, China

**Keywords:** *Camellia oleifera*, Light-emitting diode, Light quality, Proliferation, In vitro culture

## Abstract

**Background:**

*Camellia oleifera* is an important oil-yielding woody plant native to China. Tea oil extracted from the seeds is rich in health-beneficial compounds. Huajin is a high-yielding elite variety of *C. oleifera*, with large fruits and remarkable resilience, widely cultivated in southern China; however, its seedling quality tends to be uneven. At present, techniques such as grafting, and cuttings are primarily adopted to propagate *C. oleifera*. These approaches are susceptible to environmental constraints owing to the long growth period, resulting in the lack of *C. oleifera* seedlings. Methods to make the cultivation more economical are warranted; this can be facilitated by tissue culture technology to provide good-quality seedlings in a short time.

**Methods:**

In vitro cultured plantlets of *C. oleifera* Huajin were exposed to red light (RL), blue light (BL), red:blue light at a 4:1 ratio (R4:B1), and red:blue light at a 1:4 ratio (R1:B4); white light (WL) was used as the control treatment. To investigate the influence of light spectral quality on the proliferation coefficient, photosynthetic pigments, soluble proteins, plant height, leaf shape, Rubisco enzyme activity, and stomata and leaf anatomical features.

**Results:**

The highest proliferation coefficient was observed under combined red and blue (4:1) light. In addition, this treatment resulted in the second highest chlorophyll content, the thickest palisade and spongy tissues, and consequently, the thickest leaves. The same treatment resulted in the second highest stomatal density, albeit concomitantly with the smallest average stomatal length and width.

**Discussion:**

These results indicate that high-quality propagation of Huajin shoots can be achieved by culturing the plants in vitro under a combination of red and blue (4:1) lights. Previous studies have shown that red and blue lights improve rooting and transplanting rates of tissue culture seedlings. Hence, future research should focus on the effect of light quality on rooting and transplanting of tissue culture plantlets of Huajin and its specific molecular mechanisms.

## Introduction

*Camellia oleifera* (family, Theaceae) is an important oil-yielding, woody evergreen shrub or small tree native to China (Gao, Yang & Yuan, 2017). The four major trees producing edible oil in the world are *C. oleifera*, olive (*Olea europaea*), oil palm (*Elaeis guineensis*), and coconut (*Cocos nucifera*) (Li et al., 2016). The main product of *C. oleifera* is tea oil, also known as ‘Oriental olive oil’, which is highly recommended as a healthy edible oil by the International Food and Agriculture Organization. It is rich in unsaturated fatty acids, squalene, vitamin E, and other nutrients (Li et al., 2016). Tea oil is known to decrease lipid concentrations and prevent hypertension and the hardening of arteries (Li et al., 2016). Huajin is a high-yielding elite variety of *C. oleifera* widely cultivated in southern China, having large fruits and remarkable resilience. However, the main propagation techniques used are grafting and cuttings, which are susceptible to environmental constraints owing to a long growth period, resulting in the lack of *C. oleifera* seedlings and uneven quality. In vitro propagation can be used to obtain many high-quality shoots in a short time. Therefore, plant tissue culture techniques can be of great significance to breeding efforts committed to obtaining outstanding *C. oleifera* varieties. Studies have yielded varieties of *C. oleifera* and sterile tissue-cultured plantlets by using the tissue culture technique (Li et al., 2016). However, these studies showed low multiplication rates for adventitious buds and several months for regeneration. Moreover, there have been no studies on the tissue culture of the variety Huajin. To meet the demand for tea oil, it is necessary to scale up production by developing tissue culture technologies for the large-scale propagation of *C. oleifera* trees in south China.

Light is a key environmental factor affecting almost every aspect of plant life. It is the primary source of energy for photosynthesis and serves as a developmental cue to harmonise growth with the ambient light environment, namely photomorphogenesis (Viczian et al., 2017). Light affects the growth and physiology of plants in a complex way (Olle & Viršile, 2013). Plants have sophisticated photosensitive mechanisms to capture light energy for photosynthesis (Walters, 2005); they can sense even slight changes in light energy via cryptochrome and phytochrome receptors and make the corresponding changes (Wang & Folta, 2013). The quality of light affects photosynthesis and other developmental and biochemical processes, such as plant shape (Singh et al., 2015), germination (Taylor & Assmann, 2001), flowering (Hahn et al., 2006), and stomatal regulation (Taylor & Assmann, 2001), but different plant species respond differently to light quality (Alvarenga et al., 2015). For example, the combination of blue and red (3:1) spectra was more suitable for in vitro cultured rapeseed (Li, Tang & Xu, 2013), and the red/blue (1:1) combination was more suitable for *Vaccinium corymbosum* (Hung et al., 2016). Previous studies have indicated that light has a positive effect on growth and accumulation of plant secondary metabolites (Wang et al., 2018). Wang et al. (2018) found that blue light supplementation positively influenced *A. roxburghii* secondary metabolite accumulation.

Maximising lighting precision for optimal plant growth significantly improves photosynthesis and ultimately, crop yield (Yang et al., 2018). Currently, tissue culture techniques are routinely used in plant science and many commercial applications. One way to improve light quality for plant tissue culture is the use of light-emitting diodes (LEDs) as light sources. LEDs are considered the most economical and potent available light sources for this purpose owing to the ease of their installation and maintenance (Yang et al., 2018). Furthermore, they are durable and space-saving and produce light of specific wavelengths at high luminous flux and low radiant heat, which can optimise light distribution and reduce heat in incubators and greenhouses (Singh et al., 2015). LED lamps with different wavelengths (<400 nm, ultraviolet; 400–450 nm, violet; 450–500 nm, blue; 500–570 nm, green; 570–590 nm, yellow; 590–610 nm, orange/amber; 610–760 nm, red; and >760 nm, infrared) can be used alone or combined to optimise photosynthesis (Singh et al., 2015; Shengxin et al., 2016). Thus, LEDs of different wavelengths can be used individually or in combination to optimise photosynthesis (Singh et al., 2015; Shengxin et al., 2016). All these advantages have made LEDs the preferred source for regulating plant growth in tissue culture environments (Alvarenga et al., 2015).

Appropriate light quality can effectively improve the growth of tissue culture seedlings, but the effects of light quality on the proliferation of Huajin shoots in tissue culture have not been studied. Thus, the hypothesis of this study was that different LED light qualities have different effects on the growth of *C. oleifera*. This study aimed to use energy-saving LED lamps to explore the effects of different light qualities on the proliferation coefficient, photosynthetic pigments, soluble proteins, shoot height, leaf shape, Rubisco enzyme activity, and stomata and leaf anatomical features in the multiplication stage of *C. oleifera*, and to determine the best quality light for optimum propagation.

## Materials & Methods

### Plant materials and growth conditions

The experiment was performed in 2018 at the Key Laboratory of Cultivation and Protection for Non-Wood Forest Trees, Ministry of Education, at the Central South University of Forestry and Technology, Changsha, China (28°05′N, 113°21′E). Adventitious shoots from the axillary buds of Huajin were obtained from the Key Laboratory of Cultivation and Protection for Non-Wood Forest Trees as the experimental material. Adventitious shoots from the axillary buds were cultured in $ \frac{1}{2} $ Murashige and Skoog medium + 3.0 mg L^−1^ 6-benzylaminopurine + 0.02 mg L^−1^ indole butyric acid + 30 g L^−1^ sucrose + 7 g L^−1^ agar, under different light conditions. Each bottle has three adventitious shoots.

**Figure 1:**
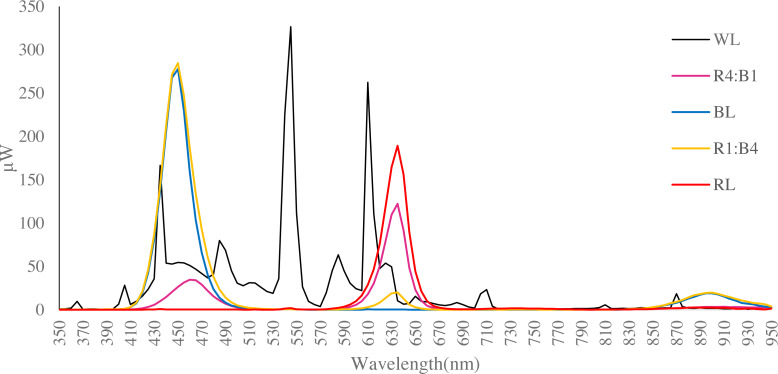
Spectral distribution of LED light sources utilised (data obtained using a Hopocolor OHSP-350SF Spectral Colour Luminance Meter). Each line indicates the wavelength of the test light quality. WL is white light, R4:B1 is red:blue ratio is 4:1; BL is blue light, R1:B4 is red-blue 1:4; RL is red light.

Adventitious shoots were subjected to five different light quality treatments: red light (RL), blue light (BL), red and blue lights combination (red:blue) at a 4:1 ratio (R4:B1) and 1:4 (R1:B4) ratio; white light (WL) was used as the control treatment. The spectral characteristics of the lamps are shown in Fig. 1, as measured by a Hopocolor OHSP-350SF Spectral Colour Luminance Meter. The LED lamps were placed above the tissue cultured shoots. The height of each light fixture was adjusted to ensure the light intensity was 50 ± 5 µmol m^−2^ s^−1^ (light intensity was measured with the same Hopocolor Luminance Meter). Different treatments were insulated from one another by black shading materials. For each treatment, 30 bottles of adventitious shoots were used, and each experiment was repeated thrice. All the treatments were set under a 16 h photoperiod (6:00 am to 10:00 pm), and cultures were maintained at 27 °C ± 1 °C. All tissue cultured plants were incubated under these conditions for 30 days before further analyses.

### Biomass and growth parameter analysis

After 30 days of treatment, three bottles of tissue culture shoots were chosen randomly from each treatment for subsequent testing. The proliferation coefficient was calculated as the number of adventitious shoots after inoculation divided by the number of adventitious shoots before inoculation. The length, width, and area of the third leaf from the top in each plantlet, as well as the tallest shoot from the base of the shoot to the top of the shoot, were measured by vernier callipers. The total number of all unfolded leaves of each shoot was counted. All measurements were performed thrice.

### Chlorophyll content

After 30 days of treatment, nine tissue culture seedlings were chosen randomly from each treatment for subsequent testing. Chlorophyll and carotenoid contents were determined using the method of Lichtenthaler (Harmut, 1987). Fresh mature leaves (0.1 g) were collected from each treatment for the determination of chlorophyll (Chl a, Chl b, Chl a + b) and carotenoid contents. Samples were cut into small pieces. Chlorophyll and carotenoid were extracted in 10 mL acetone–absolute ethanol solvent (2/1, v/v) in the dark at room temperature. Chlorophyll and carotenoid quantification were performed at 663, 645, and 470 nm. The results were expressed as milligrams of chlorophyll and carotenoid mass per gram of fresh weight. All measurements were performed thrice.

### Soluble protein content and Rubisco enzyme activity

Soluble protein content in macerated fresh samples was determined using the assay kit A054-2 (Nanjing Jiancheng Bioengineering Institute, Nanjing, China) according to manufacturer instructions. A total of 0.1 g of fresh leaf sample was ground in a mortar with 10 mL PBS. The optical density was measured at 595 nm.

Rubisco enzyme activity in macerated fresh samples was determined using the BC0440 assay kit (Beijing Solarbio Science and Technology Co., Ltd., Beijing, China) according to manufacturer instructions. Fresh leaves (0.1 g) were homogenised in 1 mL of extract in an ice bath and centrifuged at 10,000 × g and 4 °C for 10 min. The supernatant was taken and placed on ice for testing. The optical density was measured at 340 nm. All measurements were repeated thrice.

### Stomata observations

The third leaf from the top in each shoot was collected from each treatment after a 30-day culture. Absorbent cotton fibre was wet with water and used to wipe the abaxial and adaxial surfaces of the leaves. After the leaves were dried, transparent nail enamel was smeared on both sides approximately 0.5 cm from the main vein and allowed to air dry. A transparent adhesive tape was pressed onto the leaf and subsequently peeled off to obtain epidermal prints (Bin, Qingya & Canming, 2008), and a 200 µm × 200 µm area of the peel was projected via a microscope onto a flat surface. The resulting images were analysed using ImageJ software (version 1.48V; NIH, USA). The number of stomata in 30 such 200 µm × 200 µm areas was expressed as the number of stomata per 40,000 µm^2^ (stomatal density). The length, width, perimeter, and area of randomly selected stomata were measured under 40X magnification. Thirty stomata were measured per treatment.

### Leaf anatomical features

The third leaf of each of 10 tissue culture shoots was collected as above, and the middle one-third of the leaf segment was fixed with formalin-alcohol-acetic acid, stored in 70% alcohol, dehydrated, embedded in transparent medium, and then sliced to 10 µm sections using a microtome. The sections were stained with crocus solid green. ImageJ software was used to determine the thickness of the leaf, palisade tissue, spongy thin-walled tissue, and epidermis.

### Statistical analysis

Data collection and statistical analyses were performed using Microsoft Excel (2007) and IBM SPSS Statistics for Windows, version 19 (IBM Corp., Armonk, NY, USA). The results were analysed by one-way analysis of variance (least significant difference test).

## Results

### Morphological observations

**Figure 2:**
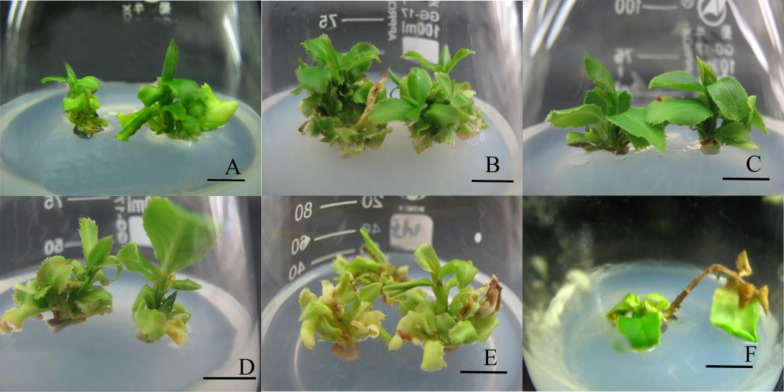
Growth of Huajin tissue-cultured plantlets under various light treatments. (A) White light (WL, control); (B) red and blue (R4:B1); (C) blue light (BL); (D) red and blue (R1:B4); (E, F) red light (RL). Scale bar = 1 cm.

The growth indices of *C. oleifera* exposed to different light treatments on day 30 were summarised in Fig. 2 and Table 1. Overall, the R4:B1 treatment made *C. oleifera* tissue culture seedlings grow robustly. Significantly higher proliferation coefficients (7.33 ± 0.11 vs. 4.68 ± 0.94, *P* < 0.01) were observed under the R4:B1 treatment, at 56.6% higher than those of the control. The shoots under WL developed slowly (Fig. 2A) but grew well under R4:B1 (Fig. 2B). Meanwhile, under BL, the base of the shoots proliferated and produced several but empty buds (Fig. 2C). In turn, under R1:B4, shoots still proliferated more at the base, although they were short (Fig. 2D). In contrast, under RL, shoots were weak, with only a few shoots, and they eventually died (Fig. 2E). The shoots under R4:B1 light were the second tallest. Conversely, under R1:B4, the shoots were the shortest (Table 1). Shoots grown under RL were significantly taller than those in the other treatment groups; however, some young shoot exhibited browning of the internodes and subsequently, whole shoots browned and died (Fig. 2F).

**Table 1:** Effect of light quality on the morphology of Huajin tissue-cultured.

Light quality	proliferation coefficient	shoot height (cm)	Growth status	Growth
WL	4.68 ± 0.94^Cd^	1.067 ± 0.51^Bbc^	+++	Good growth, more shoots, but slow growth
4R:1B	7.33 ± 0.11^Aa^	1.54 ± 0.67^ABab^	++++	Good growth, many buds, fast growth
BL	5.04 ± 0.55^Cc^	1.24 ± 0.52^ABb^	++	Normal growth, more buds, average growth
1R:4B	5.68 ± 0.12^Bb^	0.81 ± 0.46^BC^	++++	Good growth, more shoots, faster growth
RL	2.42 ± 0.89^De^	1.69 ± 0.54^Aa^	+	Poor growth, few buds, slow growth

**Notes.**

The more + symbols, the greater the shoot growth. Values are means ± SE. Different uppercase letters indicate highly significant difference (*P* < 0.01) and lowercase letters indicate a significant difference (*P* < 0.05), based on one-way ANOVA followed by the LSD test.

Significantly higher leaf numbers (14.5 ± 0.70 vs. 8.2 ± 0.49, *P* < 0.01) were observed under the 4R:1B treatment, at 76.8% higher than those of the control (Table 2). However, a significantly higher leaf area was obtained for the WL (control) treatment, with the leaves having higher values for both length (352.60 ± 19.57 mm) and width (16.88 ± 0.75 mm). In contrast, significantly lower leaf area was obtained for the 4R:1B treatment, with the leaves having lower values for both length (19.15 ± 0.94 mm) and width (12.11 ± 0.39 mm).

**Table 2:** Effect of light quality on leaves of tissue cultured seedlings.

Light source	Leaf length (mm)	Leaf width (mm)	Leaf area (mm^2^)	Leaf numbers
WL	26.32 ± 1.32^Aa^	16.88 ± 0.75^Aa^	352.60 ± 19.57^Aa^	8.2 ± 0.49 Bc
4R:1B	19.15 ± 0.94^Bb^	12.11 ± 0.39^Bc^	183.86 ± 16.40^Cc^	14.5 ± 0.70 Aa
BL	24.00 ± 0.91^ABab^	14.32 ± 0.45^Bb^	278.11 ± 14.58^Bb^	12.3 ± 0.73 Ab
1R:4B	20.16 ± 1.00^Bb^	13.09 ± 0.81^Bb^	207.92 ± 21.85^Cc^	12.6 ± 0.86 Aab
RL	21.66 ± 0.55^Bb^	13.51 ± 0.40^Bb^	219.06 ± 7.48^BCc^	7.9 ± 0.57 Bc

**Notes.**

Values are means ± SE. Different uppercase letters indicate highly significant difference (*P* < 0.01) and lowercase letters indicate significant difference (*P* < 0.05), based on one-way ANOVA followed by the LSD test.

### Photosynthetic pigment contents

Total chlorophyll, chlorophyll a, chlorophyll b, and carotenoid contents were the highest under WL, followed by R4:B1, while the corresponding values under R1:B4 were the lowest (Table 3). Leaf pigment content was significantly higher under WL than under any other treatment (*P* < 0.05), and it was significantly lower under R1:B4. Monochromatic BL treatment resulted in the highest chlorophyll a/b ratio, followed by WL, but there was no significant difference between the two. The RL and R1:B4 treatments showed the lowest chlorophyll a/b ratios, which were significantly lower than under any other treatment (Table 3).

**Table 3:** Effect of light quality on physiological indexes of tissue culture seedlings.

Light source	WL	4R:1B	BL	1R:4B	RL
Chlorophyll a (mg/g FW)	1.32 ± 0.06^Aa^	0.95 ± 0.13^Bb^	0.79 ± 0.11^Bbc^	0.61 ± 0.14^Bc^	0.81 ± 0.19^Bbc^
Chlorophyll b (mg/g FW)	0.48 ± 0.05^A^	0.34 ± 0.01^B^	0.22 ± 0.13^D^	0.27 ± 0.01^C^	0.32 ± 0.03^B^
Carotenoids (mg/g FW)	0.23 ± 0.11^Aa^	0.21 ± 0.12^ABa^	0.16 ± 0.14^Bb^	0.13 ± 0.08^Bb^	0.15 ± 0.01^Bb^
Total chlorophyll (mg/g FW)	1.78 ± 0.55^Aa^	1.29 ± 0.10^Ab^	1.05 ± 0.87^Bb^	0.85 ± 0.10^Bc^	1.121 ± 0.16^Bb^
Chlorophyll a/b	2.85 ± 0.78^ab^	2.81 ± 0.61^b^	3.04 ± 0.47^a^	2.56 ± 0.87^c^	2.59 ± 0.50^c^
Soluble protein (gprot/L FW)	4.96 ± 0.44^B^	6.64 ± 0.40^A^	5.89 ± 0.82^B^	5.60 ± 0.37^B^	5.96 ± 0.49^B^
RuBisCO (U/g FW)	74.00 ± 2.18^A^	44.97 ± 2.52^C^	80.45 ± 1.19^A^	26.17 ± 2.68^D^	60.24 ± 3.56^B^

**Notes.**

Values are means ± SE. Different uppercase letters indicate highly significant difference (*P* < 0.01) and lowercase letters indicate significant difference (*P* < 0.05) based on one-way ANOVA and the LSD test.

### Soluble protein content and Rubisco activity

Soluble protein content was significantly higher under R4:B1 than under any other treatments, although no significant differences were observed. Soluble protein content was ranked as follows: R4:B1 >RL >BL >WL >R1:B4 (Table 3).

Rubisco activity was higher in plants cultured under BL than under WL, but there was no significant difference between the two treatments. The lowest activity was observed in plants cultured under R1:B4, with only 32.5% of the activity recorded for BL-treated plants.

### Stomatal observation

**Figure 3:**
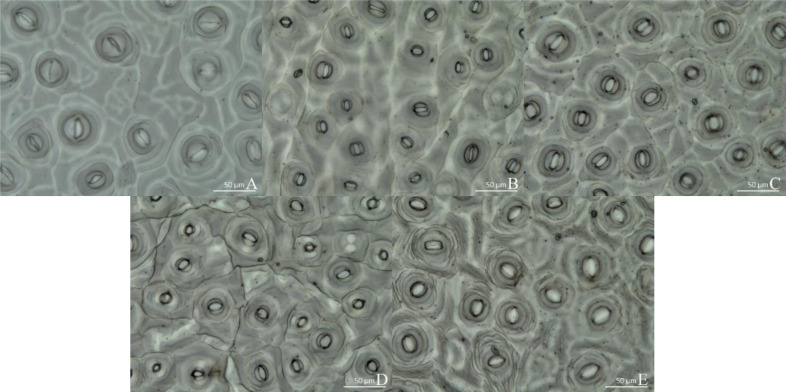
Stomatal growth under various light treatments. (A) White light (WL); (B) red:blue (R4:B1); (C) blue light (BL); (D) red:blue (R1:B4); (E) red light (RL). Scale bar = 50 µm.

The micrographs in Fig. 3 reveal that the stomatal density and morphology varied with light spectral quality. Thus, under WL, stomata were long and narrow (Fig. 3A), whereas under RL and BL treatments, they were short and round (Figs. 3B and 3C). The stomata under RL were also long and narrow, but with larger apertures than those under WL. In all the treatments, the stomata had accessory guard cells, and they were open. Among all the treatments, the R1:B4 treatment resulted in the highest stomatal density, at approximately 10.9 per 40,000 µm^2^, which was significantly higher than that under WL or BL. There was no significant difference in stomatal density between the R4:B1 treatment and R1:B4 (Table 4). Under R4:B1, the stomata were significantly smaller than those under any other treatment. The stomata under the RL, BL, and R1:B4 treatments were significantly wider than those under WL and R4:B1. The aspect ratio of the stomata under R1:B4 was the smallest and thus most circular; this was significantly different from the aspect ratio under the other treatments. The stomatal aspect ratio was the largest under WL, whereas the stomatal area was significantly smaller under R4:B1 than under any other treatment; conversely, stomatal area under RL was the largest among all treatments under evaluation.

**Table 4:** Effect of light spectral quality on stomatal characteristics.

	WL	4R:1B	BL	1R:4B	RL
Stomatal Density (number per 40,000 µm^2^)	8.7 ± 0.22^C^	10.5 ± 0.19^AB^	10.0 ± 0.20^B^	10.9 ± 0.27^A^	7.7 ± 0.15^D^
Length (µm)	19.37 ± 0.32^Aa^	14.30 ± 0.41^Cc^	16.17 ± 0.48^Bb^	15.70 ± 0.54^BCb^	19.69 ± 0.38^Aa^
Width (µm)	5.06 ± 0.38^B^	5.15 ± 0.21^B^	8.23 ± 0.37^A^	8.26 ± 0.35^A^	9.10 ± 0.38^A^
Aspect ratio	3.06 ± 0.46^A^	2.37 ± 0.30^B^	1.76 ± 0.34^C^	1.63 ± 0.48^C^	1.74 ± 0.44^C^
Circumference (µm)	42.51 ± 0.61^B^	31.40 ± 0.82^D^	41.60 ± 1.00^B^	35.22 ± 1.04^C^	49.02 ± 0.89^A^
Area (µm^2^)	95.07 ± 0.56^C^	60.32 ± 0.86^E^	113.75 ± 0.88^B^	88.00 ± 0.95^D^	164.61 ± 0.92^A^

**Notes.**

Values are means ± SE. Different uppercase letters indicate highly significant difference (*P* < 0.01) and lowercase letters indicate significant difference (*P* < 0.05) based on one-way ANOVA and LSD test.

### Leaf structure

**Figure 4:**
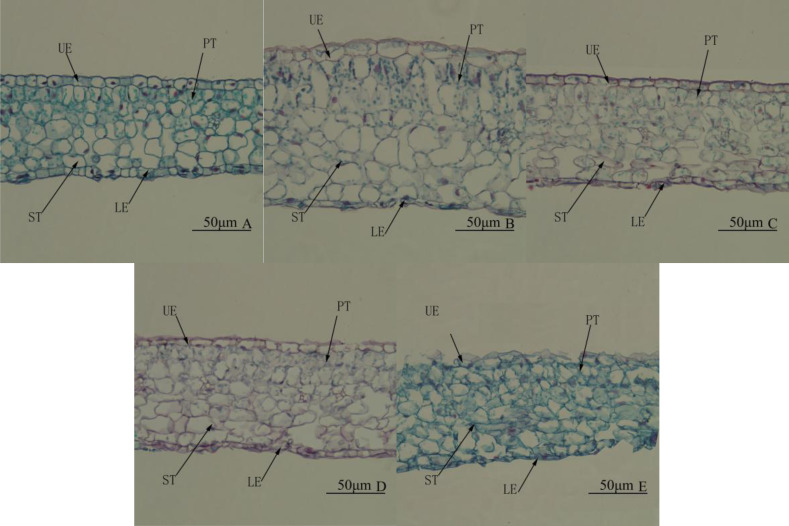
Leaf anatomy. Cross-section of the middle part of the leaf blade of *Camellia oleifera* cultured under light of different spectral quality, photographs were taken at 20 × magnification. Photographs were taken at 20 × magnification. (A) White light (WL); (B) red:blue (R4:B1); (C) blue light (BL); (D) red:blue (R1:B4); (E) red light (RL). UE, upper epidermis; LE, lower epidermis; PT, palisade mesophyll tissue; ST, spongy mesophyll tissue. Scale bar = 50 µm.

Leaf structure differed significantly among treatments. Under WL, there were two layers of palisade tissue arranged closely and in an orderly manner. The spongy tissue was significantly larger than palisade tissue and looked more compact (Fig. 4A). Under R4:B1, the leaves were the thickest, mainly because palisade and spongy tissues were significantly thicker than under any other treatment (Table 5 and Fig. 4B). Under BL, the lower epidermis was the thickest, and the leaves were the second thickest (Table 5). Under RL, all tissues were smaller than that under any other treatment; the palisade tissue was not obvious, and the spongy tissue appeared disordered (Table 5 and Fig. 4E). In all treatments, the upper epidermis was thicker than the lower epidermis, and the spongy tissue was thicker than the palisade tissue.

**Table 5:** Effect of light quality on leaf anatomy.

Treatment	Thickness of upper epidermis/ µm	Thickness of lower epidermis/ µm	Thickness of palisade parenchyma/ µm	Thickness of spongy Parenchyma/ µm	Thickness of blade/µm
WL	12.51 ± 0.36^Aa^	8.49 ± 0.45^A^	29.45 ± 0.40^Cd^	56.72 ± 1.10^Dd^	108.84 ± 1.22^Ccd^
4R:1B	13.00 ± 0.60^Aa^	7.57 ± 0.39^A^	47.21 ± 1.65^Aa^	88.59 ± 2.45^Bb^	150.29 ± 1.19^Aa^
BL	9.20 ± 0.29^Bb^	7.67 ± 0.35^A^	33.59 ± 0.73^Cc^	78.51 ± 0.98^Aa^	123.04 ± 2.01^Bb^
1R:4B	8.76 ± 0.39^Bbc^	4.79 ± 0.20^B^	39.71 ± 1.22^Bb^	62.75 ± 1.62^CDc^	113.12 ± 1.61^Cc^
RL	7.80 ± 0.43^Bc^	5.15 ± 0.29^B^	31.33 ± 1.34^Ccd^	67.30 ± 1.67^Cc^	107.72 ± 1.35^Cd^

**Notes.**

Values are means ± SE. Different uppercase letters indicate highly significant difference (*P* < 0.01) and lowercase letters indicate significant difference (*P* < 0.05), based on one-way ANOVA and the LSD test.

## Discussion

Light quality is a key environmental factor affecting plant growth mainly via its influence on photosynthesis (Wang et al., 2016; Manivannan et al., 2017; He et al., 2019). Photosynthesis is strongly influenced by red, blue, and red plus blue colours of LED lamps, because they are the major energy sources for photosynthetic CO_2_ assimilation in plants (wavelengths between 400 and 700 nm) (McCree, 1971). Under micropropagation, plants are often exposed to conditions characterized by high relative humidity inside the vessels and low photosynthetic active radiance. Improvement of light quality, such as using LED lights, can be exploited to improve photosynthetic performance. Li, Xu & Tang (2010) speculated that mixing certain blue and red LED light sources could combine the advantages of monochromatic red and blue LEDs, overcoming their disadvantages and promoting plant growth. However, the ideal wavelength proportions can vary with plant species. Supplementing BL together with RL can reportedly increase photosynthetic rate and plant biomass significantly (Ma et al., 2001; Kim et al., 2004a).

### Biomass parameter response of *C. oleifera* Huajin to light quality

Increasing the proliferation coefficient under the premise of ensuring the quality of tissue culture seedlings is an effective way to reduce the cost of production per plant and increase production efficiency. It is also one of the criteria for factory production of tissue culture seedlings. Appropriate LED light can increase the proliferation coefficient of tissue culture seedlings, but the light quality conditions required by different plants differ. In this study, R4:B1 light produced the highest proliferation coefficient in tissue cultured shoots of Huajin, and the growth of tissue culture shoots was the best; similar findings were obtained for a species of rose (Azmi, Ahmad & Ibrahim, 2014). However studies on *Chrysanthemum* (Kim et al., 2004b) and *Abeliophyllum distichum* (Lee, Choi & Moon, 2014) showed that the proliferation of plants cultured under red:blue (1:1) light was the highest among treatments tested, while that of the rapeseed cultivar Westar was relatively high under BL (Li, Tang & Xu, 2013). These results indicate that the effects of light quality on proliferation coefficient vary according to the plant species. LEDs can be used to improve shoot-root inductions during in vitro culturing (Budiarto, 2010), therefore, this study provided basis for further investigation into the effect of light quality on rooting of *Camellia oleifera* tissue culture seedlings.

In this study, although RL produced the tallest shoots in Huajin, the proliferation coefficient was the lowest, and the internodes of the plants showed progressive browning during later stages of growth, followed by plant death. Similar results were reported for lettuce (Yanagi, Okamoto & Takita, 1996), banana (Aksenova et al., 1994), *Camellia sinensis* Huangjinya (Tian et al., 2019), and *Plectranthus amboinicus* (Silva et al., 2017), the reason being that light is perceived by plants through photoreceptors, such as phytochromes and cryptochromes, which generate a series of specific physiological responses (Taiz et al., 2017). The production of kaempferol derivatives by plants in response to RL might support apical dominance, thus causing plant stem elongation (Chée, 1986). However, it is believed that monochromatic RL causes an imbalance in light energy distribution available for optimal functioning of photosystems I and II (Tennessen, Singsaas & Sharkey, 1994), resulting in elongated and fragile stems and reduced plant biomass, ultimately affecting plant growth (Mengxi et al., 2011). Kim concluded that, depending on the synergistic interaction between blue/red light receptors and photosensitizing pigments, stem elongation might be either promoted or inhibited to varying degrees (Kim et al., 2004b). In this study, red-blue mixed light reduced the disadvantage of RL alone.

In our study, WL resulted in the highest leaf length, width, and area in tissue-cultured plantlets of Huajin, but the leaf numbers were not the largest. This is consistent with the findings in Manicure Finger grapes (Li et al., 2017). However, different plants have different responses to light quality. The leaf area of *C. sinensis* Huangjinya was found to be the largest under BL (Tian et al., 2019). In tissue cultured seedlings of *Plectranthus amboinicus*, the leaf area was the highest under RL and red:blue (1:1) treatments, whereas it was the lowest under BL (Silva et al., 2017). Exposure to the 4R:1B treatment significantly increased leaf numbers. Although the area of a single leaf was smaller than that of the WL control, the total area of leaves of Huajin tissue cultured seedlings was higher than in the control. This might also be one of the reasons for the best growth state of Huajin in vitro cultured seedlings under red and blue (4:1) light.

### Photosynthetic pigment response of *C. oleifera* Huajin to light quality

Chlorophyll is a pigment responsible for light absorption during photosynthesis, and its concentration and composition directly influence leaf photosynthetic rate (Fan et al., 2013). Chlorophyll a mainly absorbs red-orange light (640–660 nm), while chlorophyll b mainly absorbs blue-violet light (430–450 nm). In this study, the chlorophyll content in Huajin tissue-cultured plantlets was relatively high under WL or R4:B1. Kim also found that the chlorophyll content in *Chrysanthemum* plants grown in vitro under red-blue mixed light was the highest among other light types (Kim et al., 2004b). Chlorophyll content also increased in *Staphylea* grown under red-blue mixed light (Szewczyk-Taranek et al., 2015), likely due to an increased sensitivity of photosensitive pigments to RL and changes in the synthesis of chlorophyll and other pigments induced by BL through genetic regulation (Gupta & Jatothu, 2013; Liu et al., 2014). However, the effects of light spectral quality are related to spectral irradiance and plant species (Batista et al., 2018); therefore, different plants require different red-blue light ratios. Some studies have shown that plant leaves absorb approximately 90% of RL and BL (Terashima et al., 2009), and the spectral energy distribution of RL and BL is consistent with that of chlorophyll absorption (Goins et al., 1997). Thus, the mixture of RL and BL could enhance plant growth and development by increasing net assimilation (Tanaka et al., 1998). This might have increased the chlorophyll content, enhanced growth, and increased the proliferation coefficient in tissue cultured Huajin plantlets at an appropriate red-blue mixed light ratio. In our study, the R4:B1 treatment produced the largest total area of leaves, indicating a high total chlorophyll content. The chlorophyll a/b ratio is positively related to the capacity for electron transport and Calvin cycle enzyme activities. Less chlorophyll b includes a highly adaptive combiner with light harvesting antenna, thus having higher electronic transmission capacity (Evans, 1988). The R4:B1 treatment also resulted in a rather high chlorophyll a/b ratio, which may also be one of the reasons for the best growth.

Carotenoids play vital roles in photosynthesis by absorbing light, protecting chlorophyll from photo-oxidation; attracting pollinators and seed dispersers by imparting colour to the leaves, flowers, and fruits; serving as precursors of compounds such as abscisic acid and vitamin A (Carvalho, Fraser & Martens, 2013; Tuan et al., 2013). As photosensitive pigments, carotenoids are susceptible to photo-oxidation; they absorb in the 450–550 nm region of the light spectrum not used by chlorophyll a or b and can rapidly extinguish chlorophyll excitation, thus protecting photosystems PSI and PSII from photo-oxidation stress (Hashimoto, Uragami & Cogdell, 2016). They can also assist in the transformation of inorganic molecules or ions into organic biomolecules (Yu et al., 2017). In our study, the carotenoid content was the highest under WL and R4:B1. This is consistent with the results of Tuan et al. (2013) in *Fagopyrum tataricum*. The study also reported that carotenoid biosynthetic pathway-related gene transcription was higher under WL than under BL and RL. The expression of these mRNAs may lead to a high content of carotenoids in seedlings in the later period of growth under WL (Tuan et al., 2013). The protective and transformative roles of carotenoids may explain why our cultured plants showed the highest proliferation coefficient under the R4:B1 treatment.

### Rubisco activity and soluble protein response of *C. oleifera* Huajin to light quality

Rubisco is a key enzyme in plant photosynthesis. In addition to controlling the fixation of CO_2_, the enzyme restricts the flow of carbon to the Calvin and photorespiration cycles. The activity of Rubisco directly affects the photosynthetic rate. In this study, the activity of Rubisco was the highest under BL, which is consistent with the results obtained in soybean plants (Eskins, Jiang & Shibles, 1991). This might be because the transcription of the small subunit of Rubisco is upregulated by BL (Sawbridge et al., 1993). In cucumber seedlings, BL reportedly increases stomatal conductance and Rubisco activity significantly, further upregulating the transcription of genes encoding Calvin cycle enzymes and reducing the CO_2_ assimilation rate (Wang et al., 2009). This may partially account for the mid-range proliferation coefficient seen in the BL-treated plants in our study, despite their high Rubisco activity.

Soluble proteins are important molecules that act as osmotic regulators protecting living matter in cells and biofilms. Wang speculated that Rubisco was the main component of leaf soluble protein (Wang et al., 2009). Our results showed that the soluble protein content was high under BL, which is consistent with this view, although R4:B1 resulted in the highest soluble protein content. Moreover, the R4:B1 treatment showed the highest proliferation coefficient, likely because the Rubisco enzyme activity in Huajin leaves was not the highest under 4R:1B, but the utilization rate was high. RL may ensure the efficiency of CO_2_ assimilation, while other soluble proteins provide more nutrients for plant development and promote proliferation.

### Stomata response of *C. oleifera* Huajin to light quality

Stomata help plants in adjusting to short-term environmental changes (Do Nascimento Vieira et al., 2015). By controlling the pore aperture, stomata maximise plant homeostasis by modulating the extent of physical exchange between the plant and its surroundings (Zeiger, 1984). Light quality can affect stomatal development, mediated by cryptochromes and phytochromes (Kang et al., 2009). Spectral quality can also affect the differentiation of proto-epidermal cells into stomata through the photoreceptors *cry1*, *cry2*, *phyA*, and *phyB*, which transduce BL, RL, and far RL (Kang et al., 2009). Higher stomatal density can promote the utilization of CO_2_. In our study, the R1:B4 treatment resulted in the highest stomatal density, followed by R4:B1, while RL resulted in the lowest. Therefore, combined RL and BL promoted increased stomatal density as did BL alone, whereas RL alone was inhibitory. Similar results have been reported in cherry tomato (XiaoYing et al., 2011). Our results also showed that stomata were longer and narrower under WL than under other light conditions and were the smallest under R4:B1.

### Leaf structure response of *C. oleifera* Huajin to light quality

Light spectral quality strongly affects many plant anatomical, physiological, morphological, and biochemical parameters (Sæbø, Krekling & Appelgren, 1995; Weston et al., 2000; Haliapas et al., 2008; Macedo et al., 2011). In this study, the leaf palisade and spongy tissues under R4:B1 were normally structured and significantly thicker than under any other treatment group. However, under the RL treatment, these tissues were significantly thinner than those in the other treatment groups; the palisade tissue was not obvious, and the spongy tissue was disordered. Some studies on the performance characteristic of upland cotton seedlings under different light quality treatments revealed similar results; BL treatment resulted in the highest leaf thickness and palisade tissue thickness, whereas RL resulted in the thinnest palisade tissue (Li, Xu & Tang, 2010). In cherry tomato, palisade and spongy tissue cells developed better in leaves treated with BL than those in its absence, and the leaves exposed to mixed RL-BL showed the best development (XiaoYing et al., 2011). BL was found to be important in regulating photosynthetic characteristics, resulting in enhanced production of photosynthetic pigments, and supporting the development of organelles responsible for photosynthesis (Liu et al., 2014). RL improved the sensitivity of phytochromes; thus, RL and BL together can enhance the development of palisade and spongy mesophyll cells. However, Hogewoning et al. (2010) reported photosynthetic dysfunction in cucumber seedlings under RL alone, and Macedo et al. (2011) reported that the boundary between palisade and spongy tissues in *Alternanthera brasiliana* leaves grown under RL was not clear. This is consistent with our observation of unclear palisade tissue and disordered spongy tissue under RL. Palisade tissue is reported to enable better light penetration to the chloroplasts, while spongy tissue enhances light capture by scattering light (Vogelman, Nishio & Smith, 1996; Gonçalves et al., 2008). In addition, studies have shown that the developed palisade tissue is conducive to the conduction of carbon dioxide and other gases from the lower chamber of the stomata to the photosynthetic site and can prevent water loss, thereby increasing the net photosynthetic rate. The thickest palisade and spongy tissues were found in the leaves irradiated with R4:B1 light, indicating that the net photosynthetic rate was the highest. This might also partially explain why plant proliferation was the highest among the treatment groups in this study.

## Conclusions

The R4:B1 LED had a positive effect on *C. oleifera*, which conferred a greater proliferation coefficient and markedly higher leaf numbers compared to the control treatment. Moreover, the stomatal density was the highest; the leaf cell structure was compact and neat; and the palisade tissue and spongy tissue were significantly thicker than those in other treatments. These results suggest that the R4:B1 LED is a good choice to improve the quantity and quality of *C. oleifera* seedlings. This is conducive to meeting the increasing market demand for tea oil production. In this study, we improved the propagation rate of *C. oleifera* Huajin using in vitro tissue culture. This can shorten the breeding cycle and enhance industrial development of energy-saving tissue culture techniques for this species. Future research should focus on the effect of light quality on rooting and transplanting of tissue culture plantlets of Huajin and its underlaying molecular mechanisms.

##  Supplemental Information

10.7717/peerj.10016/supp-1 Data S1 Raw data The spectral distribution of LED light sources utilised (data obtained using a Hopocolor OHSP-350SF Spectral Colour Luminance Meter) and data for all tables.

## Additional Information and Declarations

### Data Availability

The following information was supplied regarding data availability: The raw measurements are available in the Supplemental File.
